# Bifid Xiphoid Process: Case Report and Review

**DOI:** 10.7759/cureus.3153

**Published:** 2018-08-16

**Authors:** Seif Eid, Joe Iwanaga, Rod J Oskouian, Marios Loukas, R. Shane Tubbs

**Affiliations:** 1 Anatomical Sciences, St. George's University, St. George's, GRD; 2 Medical Education and Simulation, Seattle Science Foundation, Seattle, USA; 3 Neurosurgery, Swedish Neuroscience Institute, Seattle, USA; 4 Neurosurgery, Seattle Science Foundation, Seattle, USA

**Keywords:** xiphoid process, bifid xiphoid process, anatomy, variation

## Abstract

The xiphoid process is a bony process that comprises part of the sternum. This anatomical structure exhibits several morphological variations, which may complicate diagnostic examinations and invasive thoracic procedures. Variations include bifurcated or trifurcated, deflected, and curved processes. This report discusses a case of a bifid xiphoid process during cadaveric dissection and compares similar findings in the literature. The aim is to improve our knowledge of anatomical variations in an effort to reduce complications and improve patient care.

## Introduction

The xiphoid process, also referred to as the xiphisternum or ensiform process, is a bony process that is considered the most variable and smallest part of the sternum. Morphological variations range from being pointed, bifurcated, trifurcated, broad, curved, or deflected [1]. These anatomical variations are important to consider in order to prevent a misdiagnosis during radiological interventions and to prevent an iatrogenic injury during invasive thoracic procedures [2-6]. In addition to variable morphologies, the xiphoid process appears to exhibit different characteristics during development. During the early stages of life, it is cartilaginous in nature and then transforms to an ossified state in adults [1]. The xiphoid process is located in the epigastrium and serves as attachments to several muscles, including the rectus abdominis and the aponeurosis of the external and internal oblique muscles anteriorly and the diaphragm posteriorly [1].

In this report, we demonstrate a bifid xiphoid process found during cadaveric dissection, which represents one of the various anomalies described in the literature. The aim of this article is to further enhance our knowledge of anatomical variations by comparing this cadaveric finding with similar reports found in the literature.

## Case presentation

**Figure 1:**
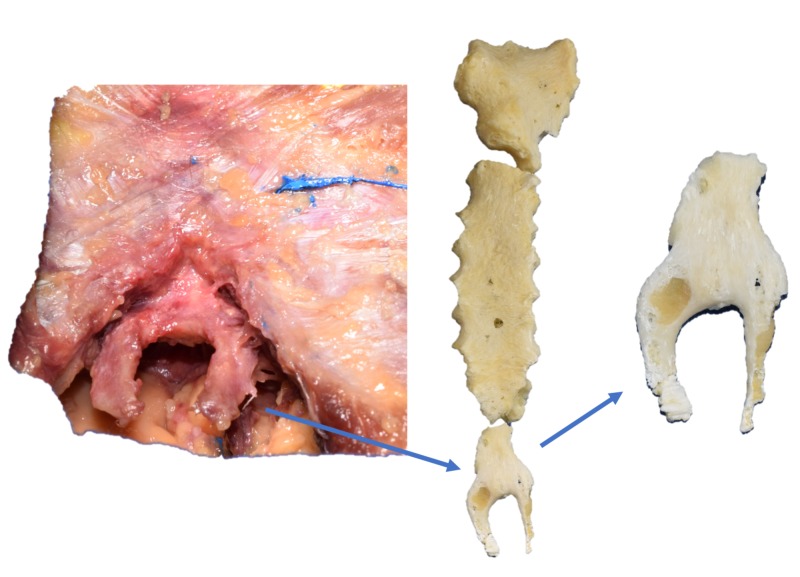
Photographs of the bifurcated xiphoid process found during dissection and after removal and cleaning

During the routine dissection of an adult male cadaver, an unusual configuration of the xiphoid process was identified. The specimen was 59 years old at death and was Caucasian. The xiphoid process was united in its upper one-half but inferiorly split into two more or less equal parts (Figure 1) of the same length of 2.2 cm. The width of each bifurcated part was 5 mm. The right part of the bifurcation was located in the coronal plane while the left part was laterally rotated about 30 degrees. The distance between the two split parts was approximately 1 cm. No vessels or nerves were identified traversing between the two parts of the bifurcated xiphoid process. Two distinct slips of the diaphragm attached to the bifurcated parts posteriorly. No other musculoskeletal or neurovascular anatomical variations were noted in the regions dissected in this specimen.

## Discussion

The variations in anatomy create a difficulty for healthcare providers to perform their roles effectively. Therefore, it is important to discuss variable anatomical findings in order to improve patient care and prevent errors in management. Variations of the xiphoid process include single ending, bifurcated or trifurcated endings, foramina, and absence [2-7]. Reports in the literature have found a variable prevalence of bifid xiphoid processes ranging from 20%-42.9%, while a single xiphoid process was found in 62.6%-80% of the cases [3-6]. Table 1 outlines the variable presentations of the xiphoid process as found in a review of the literature. Foramina through the sternum are other variants that can alter correct radiological and forensic diagnoses, and create complications during sternotomy [6,8]. A cadaveric study revealed that the xiphoid process was a common location for sternal foramina, which may contain single or multiple foramina [8]. Other cases reported the absence of the xiphoid process [3] while others reported the presence of a trifurcated xiphoid process [4-5]. In some cases, the abnormal xiphoid process could be regarded as an epigastric mass on radiographic images due to its anterior deflection [2].

**Table 1:** Occurrence of a bifid xiphoid process and other variants

Study	Type of Study	Bifid (% of cases)	Other variants
Kirum et al. [3]	Cadaveric	9 cases (42.9%)	64 cases (75.3%) with no xiphoid process
Turkay et al. [4]	Patients with imaging	125 cases (25%)	Single: 361 cases (72.2%) Trifid: 5 cases (14%) Foramina: 26 cases (5.2%)
Akin et al. [5]	Patients with imaging	164 cases (32.8%)	Single: 313 cases (62.6%) Trifid: 23 cases (4.6%) Foramina: 216 cases (43.2%)
El-Busaid et al. [6]	Cadaveric	16 cases (20%)	Single: 64 cases (80%) Foramina: 11 cases (13.8%)

## Conclusions

Anatomical variations should always be considered during diagnostic examinations and invasive procedures in order to provide ideal patient care. Knowledge of these variants is important to prevent injury and misdiagnosis. We have reported a case of a bifid xiphoid process and reviewed the literature regarding similar cases.

## References

[1] Standring S (2016). Gray’s Anatomy: The Anatomical Basis of Clinical Practice.

[2] Mashriqi F, D’Antoni AV, Tubbs RS (2017). Xiphoid process variations: a review with an extremely unusual case report. Cureus.

[3] Kirum GG, Munabi I, Kukiriza J, Tumusiime G, Kange M, Ibingira C, Buwembo W (2017). Anatomical variations of the sternal angle and anomalies of adult human sterna from the Galloway osteological collection at Makerere University Anatomy Department. Folia Morphol.

[4] Turkay R, Inci E, Ors S, Nalbant MO, Gurses IA (2017). Frequency of sternal variations in living individuals. Surg Radiol Anat.

[5] Akin K, Kosehan D, Topcu A, Koktener A (2011). Anatomic evaluation of the xiphoid process with 64-row multidetector computed tomography. Skeletal Radiol.

[6] El-Bussaid H, Kaisha W, Hassanali J, Hassan S, Ogeng’o J, Mandela P (2012). Sternal foramina and variant xiphoid morphology in a Kenyan population. Folia Morphol.

[7] Choi PJ, Iwanaga J, Tubbs RS (2017). A comprehensive review of the sternal foramina and its clinical significance. Cureus.

[8] Paraskevas G, Tzika M, Anastasopoulos N, Kitsoulis P, Sofidis G, Natsis K (2015). Sternal foramina: incidence in Greek population, anatomy and clinical considerations. Surg Radiol Anat.

